# Clinical Efficacy of Ketamine for Treatment-resistant Depression

**DOI:** 10.7759/cureus.5189

**Published:** 2019-07-22

**Authors:** Sosipatros Bratsos, Sohag N Saleh

**Affiliations:** 1 Cardiology, Imperial College London, London, GBR; 2 Pharmacology, Imperial College London, London, GBR

**Keywords:** ketamine, depression

## Abstract

Depression is a common psychiatric disorder affecting more than 300 million people worldwide. According to the Diagnostic and Statistical Manual of Mental Disorders, Fifth Edition (DSM-5), the diagnosis of depression requires at least two weeks of either low mood or anhedonia as well as four or more other symptoms such as appetite or weight changes, insomnia or hypersomnia, psychomotor agitation or retardation, loss of energy, inability to concentrate, feelings of worthlessness or excessive guilt, and suicidality. Selective serotonin reuptake inhibitors (SSRIs) target the monoaminergic system and are the commonest drugs used for treating depression, but have certain limitations, such as their delayed onset of action. Ketamine, a non-competitive NMDA receptor antagonist, has shown in several randomized controlled trials (RCTs) promising results with rapid antidepressant effects, especially in patients with severe treatment-resistant depression (TRD), which is depression that has not responded to more than two antidepressants. In this review, the clinical efficacy of ketamine in TRD has been discussed, with emphasis placed on the evidence from RCTs.

## Introduction and background

Depression is a common psychiatric disorder affecting more than 300 million people worldwide, leading to a considerably reduced quality of life and increased risk of suicide, while posing a tremendous economic burden (more than 200 billion dollars in the US) [1-4]. According to the Diagnostic and Statistical Manual of Mental Disorders, Fifth Edition (DSM-5), the diagnosis of a Major Depression Episode (MDE) requires five or more symptoms to be present within a two-week period [5]. One of the symptoms should be either a depressed mood or anhedonia. The secondary symptoms of MDE are appetite or weight changes, insomnia or hypersomnia, psychomotor agitation or retardation, fatigue or loss of energy, diminished ability to think or concentrate, feelings of worthlessness or excessive guilt, and suicidality. Currently, the most commonly used antidepressant drugs are selective serotonin re-uptake inhibitors (SSRI), which primarily target the monoaminergic system [6]. An important limitation of SSRIs is their delayed onset of action, as they take about two weeks to start having an effect and can even worsen pre-existing anxiety and suicidality during this time period, especially in younger populations [7-8]. In addition, 35% of patients fail to respond to treatment altogether [9]. Recent evidence implicates the glutamatergic system in the pathogenesis of depression and thus agents that aim to modify this system, have generated immense interest. Specifically, ketamine, a non-competitive N-methyl-D-aspartate (NMDA) receptor antagonist, has particularly been in the spotlight due to its rapid antidepressant effects and particularly in patients with treatment-resistant depression (TRD), who are significantly less likely to respond to another antidepressant and are at higher risk of functional disability [10-12]. TRD refers to depression that does not respond to more than two antidepressants [13]. The aim of this review is to discuss the clinical efficacy of ketamine for TRD, by appraising the available evidence and specifically randomized controlled trials (RCTs), which evaluated the effects of ketamine in patients with TRD.

## Review

Mechanism of action

Ketamine is thought to exert its antidepressant effects via the non-competitive antagonism of N-methyl-D-aspartate (NMDA) receptors [14]. NMDA-receptor antagonism is linked to the potentiation of glutamate transmission at α-amino-3-hydroxy-5-methyl-4-isoxazole propionic acid (AMPA) receptors and increased brain-derived neurotrophic factor (BDNF) levels, associated with antidepressant properties [15]. In mouse models, ketamine resulted in enhanced concentrations of hippocampal BDNF, while BDNF knockout mice were resistant to ketamine’s antidepressant action [16]. Another proposed mechanism is the activation of the mammalian target of rapamycin (mTOR) pathway, which has been shown in rat models to produce antidepressant effects and accelerate the formation of spine synapses in the prefrontal cortex [17].

Clinical efficacy

In a two-site, parallel-arm RCT, Murrough et. al. compared the efficacy of a single infusion of ketamine to an active placebo-control, the anesthetic midazolam, in 73 patients with TRD [18]. At 24 hours, the mean Montgomery-Åsberg Depression Rating Scale (MADRS) score was significantly lower in the ketamine compared to the midazolam group, by 7.95 points (14.77 vs 22.72; *P *< 0.001), while the likelihood of treatment response (more than 50% reduction from baseline MADRS) was significantly greater for ketamine compared to midazolam (response rates 64% and 28% respectively; *P *< 0.006). Ketamine’s benefits were maintained for up to seven days post-infusion, at which point significant differences in MADRS compared to midazolam were no longer observed. One limitation of this study was that the subject number was relatively small [19-21]. Moreover, the under-representation of ethnic minorities and the strict exclusion criteria (for example history of psychotic symptoms or substance abuse) limit the generalisability of the results, although the latter may be regarded as clinically relevant for mitigating patient risk. Additionally, treatment was restricted to otherwise medication-free subjects, which might not always occur in real-life practice and might have overemphasized ketamine’s pragmatic effects. Midazolam was chosen as an active control based on its similar pharmacokinetic properties and psychoactive effects to ketamine to enhance blinding, which is a strength of this study relative to other placebo-controlled studies [20]. Nevertheless, transient dissociative effects were comparatively high with ketamine post-drug infusion, perhaps compromising the blinding. In the future, active comparators like electroconvulsive therapy or antidepressant-antipsychotic combinations may provide better control. The unequal randomisation of patients to drug: placebo (two: one) further weakens the study’s validity, as it is possible that when outcome assessors are not agnostic about allocation (there is a higher probability that a patient will fall under the intervention group), “positive or negative events might be altered” [22]. Importantly, the follow-up period was very short, especially for a chronic and relapsing condition, like depression.

The efficacy of ketamine in sustaining its initial antidepressant effects was also evaluated in a multi-center, double-blind, placebo-controlled, parallel-group, phase two study, where 68 patients with TRD were randomized to intravenous ketamine two or three times weekly or intravenous placebo two or three times weekly for four weeks [23]. The primary outcome was the mean change in MADRS from baseline to day 15, which was significantly improved in both ketamine frequency groups compared to the respective placebo (twice weekly ketamine: −18.4; placebo: −5.7; *P *< 0.001; thrice weekly ketamine: −17.7; placebo: −3.1; *P *< 0.001). Since a significant difference in the mean MADRS score between the two ketamine frequencies was not found, a twice-weekly treatment regimen was deemed sufficient as an initial dose strategy in TRD patients, although the study lacked the power to detect significant differences between the two regimens. As with other studies, however, the treatment response rate increased beyond the first dose [21,24]. Treatment response within one week that was maintained up to day 15 was also higher in both ketamine groups compared to placebo (*P *< 0.005 and *P *< 0.004 for twice-weekly and thrice-weekly ketamine, respectively). The mean change in the MADRS score improved from baseline to day 29 for both ketamine groups but was not significantly different to placebo, which might have, however, been affected by the high dropout rate after day 15 in the placebo groups due to the lack of efficacy. The lack of active control was an important limitation, as adverse effects from ketamine may have un-blinded the active drug administration to either patients or clinicians. Furthermore, although the follow-up period was longer than most RCTs evaluating the efficacy of ketamine, this study’s duration was still short. The findings can further be critiqued due to potential industry bias, as the study was funded by the employer of the majority of the article’s authors.

In both aforementioned studies, the primary efficacy endpoints involved changes in MADRS, which despite being a widely used measure of depression, is a questionnaire and thus subjective in nature with questionable inter-rater reliability, limiting the robustness of the results [25, 26]. This problem, however, may be unavoidable in depression studies. Also, the intravenous administration of ketamine may be challenging in clinical practice, thus limiting its applicability. One RCT was identified that evaluated a non-intravenous route was by Lapidus et. al., testing the intranasal efficacy of ketamine, which was comparatively reduced relative to the intravenous administration, however [27]. Finally, the short follow-up made it unclear as to whether a full relapse would occur after the antidepressant onset, raising ethical and clinical implications [28]. Ketamine, due to its rapid onset of action, could potentially benefit those at imminent risk of suicide, as indicated by Price et. al., although the antidepressant effect diminished over time and therefore ways to prolong the initial benefit are needed [28]. One such way was thought to be the glutamate-modulator riluzole, which, however, failed to achieve relapse-prevention following ketamine infusions in two studies but perhaps cognitive behavioral therapy may be of use in maintaining ketamine’s effects [29-31].

## Conclusions

In this review, the efficacy of ketamine for treating patients with TRD was evaluated. Ketamine was shown to induce rapid antidepressant effects in patients with TRD lasting up to a week following a single intravenous infusion, while twice or thrice weekly infusions prolonged this effect for up to 15 days. However, clinical trials for ketamine in depression are still in their infancy and larger, longer-term RCTs with effective active-controls are necessary to accurately assess its efficacy. Dose optimization, alternative delivery routes, methods of relapse prevention, and the role of concomitant medication in ketamine’s antidepressant effects are only some of the questions that remain to be answered. It is also important to note that since this review focused on a specific subset of depression, namely TRD, it is unclear whether similar effects might be seen in less severe forms of depression. In summary, the use of ketamine in TRD has shown promise, yet issues regarding its applicability in clinical practice and its long-term effects ought to be addressed in the future.
